# Genotype-by-environment interaction influencing sensory attributes and biochemical components of peanuts from China

**DOI:** 10.3389/fpls.2025.1609969

**Published:** 2025-08-01

**Authors:** Yueyi Tang, Shining Cao, Chushu Zhang, Weidong Hu, Haixiang Zhou, Mian Wang, Lifei Zhu, Jing Chen, Jun Zhang, Jiancheng Zhang

**Affiliations:** ^1^ Shandong Peanut Research Institute, Shandong Academy of Agricultural Sciences, Qingdao, China; ^2^ College of Agriculture, Jilin Agricultural University, Changchun, China

**Keywords:** Arachis hypogaea L., fatty acids, flavor, G×E, oil, proanthocyanidin, protein, sucrose

## Abstract

**Background:**

Peanuts are a vital global crop and healthy food, valued for their nutritional and physiological benefits. Their sensory attributes and biochemical composition, which determine taste and nutritional quality, are influenced by multiple factors. Thirteen peanut genotypes were cultivated across three locations using a randomized block design. This study presents the first application through combined ANOVA (analysis of variance) and the GGE (Genotype and Genotype by Environment) biplot in sensory attribute evaluation research. The primary objectives are to: (1) characterize the sensory attributes of the tested genotypes; (2) analyze the genotype-environment interaction (GEI) effects; and (3) screen optimal varieties (lines) adapted to specific environmental conditions.

**Results:**

Significant differences were observed among genotypes and locations, and the G×E interactions for sensory quality and biochemical components of the tested peanuts. In GGE bioplot analysis, S9 was the best performer with crunchiness, fineness, sweetness, and overall acceptability, and L10 was the best performer in Large-seeded genotypes in overall acceptability. S9, S25, HY20, HY33, and HY20 was the best genotype at 3 locations in sucrose, oleic acid, linoleic acid, palmitic acid, and oil, respectively. Weifang (WF) demonstrated potential for producing high-quality peanuts for processing. In addition, there is an evident correlation between the biochemical components of peanut raw kernels and the sensory quality of roasted kernels. Among them, high-oleic peanuts exhibited superior crunchiness, sweetness, and roasted flavor, while high-oil varieties were less sweet with weaker flavor. High-protein peanuts were sweeter but more delicate. Proanthocyanidins positively correlated with bitterness/off-flavor and negatively with crunchiness, sweetness, and acceptability. High vitamin E reduced roasted flavor intensity.

**Conclusion:**

Genotype, environment, and their interactions significantly impact peanut sensory and nutritional traits. High oleic acid and low proanthocyanidin content are critical for desirable sensory quality in this study. Lines S9 and L10 provide genetic resources for breeding, while Weifang’s climate supports premium peanut production. This data guides peanut cultivation in similar latitudes/climates.

## 1 Introduction

The peanut (*Arachis hypogaea* L.) is not only an important crop worldwide but also an ideal food with various beneficial effects from the standpoint of nutritive and physiological function (Zhao et al., 2012). It provides a spectrum of nutrients to human population, including vegetable protein, unsaturated fatty acids, fiber, phenolic compounds, minerals, and vitamins etc (Derbyshire, 2014; Ros, 2010; Shin et al., 2009; Dean et al., 2009; Wang et al., 2008), it thus makes a significant contribution to human nutrition. Furthermore, it is worth noting that high-oleic (HO) peanuts have the potential to provide health benefits, such as reducing the risk of coronary heart disease (CHD), gallstones, and lowering cholesterol levels (Derbyshire, 2014; Ros, 2010; Bera et al., 2018; O’Byrne et al., 1997; Nawade et al., 2018). Additionally, they have been found to have beneficial effects on oxidative stress and inflammation (Ros, 2010; Nawade et al., 2018). Therefore, peanuts are increasingly recognized as a functional food.

Flavor is one of the most important quality attributes for the commercial acceptance (Miyagi, 2017), and both consumers and the peanut industry have a higher demand for peanut flavor. The peanut industrial enterprises are seeking good processing materials and the breeders are producing varieties with better flavor in response to the increasing request for peanuts. Flavor is related to its chemical composition. Proteins, free amino acids and sugars were found to be important precursors of roasted peanut flavor (Neta et al., 2010; Chiou et al., 1991; Koehler et al., 1969; Mason et al., 1969). Volatile components and concentrations affected peanut flavor as well (Schirack et al., 2006). High oleic trait could be associated with some changes in flavor or sensory quality due to their resistance to oxidation (Baker et al., 2002; Braddock et al., 1995; Mugendi et al., 1998; Pattee et al., 2002; Riveros et al., 2010; Nepote et al., 2009), only Isleib’s report contested these findings (Isleib et al., 2006). It is worth noting that the peanut protective skin, as an edible part, is rich in phenolic compounds and potentially other health-boosting compounds, and it also has an impact on the taste of peanuts (Yu et al., 2005, 2006).

In addition, the biochemical components and the flavor of peanuts can be significantly affected by many other factors: genetic factors, environmental factors, and genotype-by-environment (G × E) interaction. For the biochemical components, all effects (including G, E, and G × E) significantly contributed to variation in protein, and oil and fatty acid composition, especially the O/L ratio (Wang et al., 2024; Zhang et al., 2023; Sanders, 1982; Norden et al., 1987; Bansal et al., 1993; Dwivedi et al., 1993; Killi and Beycioğlu, 2022; Isleib et al., 2008a; Dolinassou et al., 2017; Gulluoglu et al., 2016; Bishi et al., 2013). Wang et al. evaluated G × E interaction effects on peanut seed protein, oil, and 8 kinds of fatty acids content variability (Wang et al., 2024). Sanders found that genotype and location significantly affected fatty acid composition, especially the O/L ratio from evaluating four peanut cultivars (Sanders, 1982). Norden et al. evaluated 494 genotypes in Florida for 1 year and 1 location test and found that genotype significantly affected the O/L ratio (Norden et al., 1987). Zhang et al., Bansal et al. and Dwivedi et al. reported that genotype, location, and G×E significantly contributed to variation in oil and fatty acid composition (Zhang et al., 2023; Bansal et al., 1993; Dwivedi et al., 1993). Killi et al. found significant G × E interaction effects on peanut oil and protein content (Killi and Beycioğlu, 2022). For the sensory attributes, the variation of some flavor components like sweet, bitter, astringent, fruity and roasted peanutty have been investigated (Isleib et al., 2008b; Pattee et al., 1994, 1995, 1997, 1998). Pattee et al. evaluated G × E interaction effects on sweet, bitter and roasted peanut from the 17 genotypes (including Runner, Spanish and Virginia types), and the result showed significant variation among years was observed for bitter but not for sweet (Pattee et al., 1997). Isleib et al. reported on G × E interactions for flavor attributes (sweet, bitter, astringent, fruity, etc.) of breeding lines in the Uniform Peanut Performance Test (UPPT), and found that year-by-genotype interaction was large for intensities of the roasted peanut and sweet aromatic attributes (Isleib et al., 2008b).

Genotype × environment interaction (GEI) represents a critical concern for plant breeding researchers and stands as one of the most complex challenges in breeding programs aimed at identifying high-quality and stable peanut genotypes. According to experimental conditions, there are many different methods available for evaluating the stability of genotypes. Among them, the GGE biplot (Genotype × Genotype plus Environment) method, as a graphical analysis approach, has been widely used to evaluate GEIs for yield and quality traits in regional trials. This method has been applied to investigate adaptability and stability of cultivars/genotypes in maize (Al-Naggar et al., 2021; Patel et al., 2023; Greveniotis et al., 2023), wheat (Mohamed et al., 2022; Omrani et al., 2022; Gupta et al., 2023), rice (Hasan et al., 2022; Abebe et al., 2023), sorghum (Al-Naggar et al., 2018a, b), and quinoa (Ali et al., 2018; Thiam et al., 2021; Al-Naggar et al., 2022; Anchico-Jojoa et al., 2023; Jokarfard et al., 2024).

According to the statistical data of the Food and Agriculture Organization (FAO) of 2022, the harvested area of peanuts in China is 4,459,000 hectares, second only to that of India and ranking second in the world, meanwhile, the production is 18,380,500 tons, the highest worldwide (FAO, 2022). It is important for the peanut industry to study the genotype-environment interaction of biochemical components and sensory qualities of peanuts. GGE biplots have not been previously applied to evaluate the sensory attributes and biochemical components of peanuts across diverse geographical locations, aiming to identify genotypes with optimal taste characteristics and determine “ideal” testing locations for enhancing flavor consistency among genotypes. This study presents the first application of combining analysis of variance (ANOVA) with GGE biplot methodology to determine the effect of environment, genotype, and G × E interaction on sensory attributes and biochemical components of peanuts, and to explore the relationships between biochemical components of raw peanut and sensory attributes of roasted peanuts in China. This study will provide a reference for the peanut processing enterprises in the selection of producing areas and raw peanuts with favorable sensory characteristics.

## 2 Materials and methods

### 2.1 Peanut genotypes and samples collection

Thirteen genotypes were evaluated for sensory attributes in this research, including five small-seeded genotypes (S1, S9, S11, S25, and Huayu 20) and eight large-seeded genotypes (L1, L8, L9, L10, L11, L15, L17, and Huayu 33). The control genotypes (Huayu 20 and Huayu 33) were normal oleic acid and high yield peanut varieties, while the others were high yield and high oleic acid peanuts lines (**Table 1**). The experiments were carried out in Jinzhou (JZ) and Fuxin (FX) located in Liaoning Province, as well as in Weifang (WF) located in Shandong Province. The geographical location, elevation and climatic data of the experimental locations were presented in **Table 2**. The soil types in these three locations are all sandy loam soils that are suitable for peanut cultivation. The comprehensive soil parameters regarding the physical and chemical characteristics of the experimental fields are listed in **Table 3**. All genotypes were sown under film mulch and harvested under the local standard recommended procedures of fields management for the respective locations in 2017. A randomized complete block design with 3 replications was used in this experiment. After harvesting, peanuts from all three locations were sun-dried, ensure that the moisture content of all seeds is close to 8%, then sound and mature seeds were collected for sensory evaluation.

**Table 1:** Peanut genotypes studied in this research.

Genotype	Type	Variety/Line	100-seed weight (g)	Small/Large-seeded	Growth duration (day)	Oleic acid (%)
S1	Spanish	Line	60.33	Small	120	78.05
S11	Spanish	Line	56.86	Small	120	78.31
S25	Spanish	Line	50.47	Small	120	80.49
S9	Spanish	Line	65.52	Small	120	80.45
Huayu20	Spanish	Variety	62.69	Small	120	44.79
L1	Virginia	Line	76.50	Large	126	76.73
L10	Virginia	Line	83.14	Large	126	75.37
L11	Virginia	Line	73.01	Large	126	76.38
L15	Virginia	Line	74.23	Large	126	77.50
L17	Virginia	Line	77.50	Large	126	76.21
L8	Virginia	Line	77.03	Large	126	75.58
L9	Virginia	Line	74.35	Large	126	75.98
Huayu33	Virginia	Variety	84.70	Large	126	45.35

**Table 2:** Geographical location, elevation and Climatic data for the experimental plots (Source: China Meteorological Data Service Centre, https://data.cma.cn/).

Location	Longitude	Latitude	Elevations (m)	Temperature during the growth period (°C)				Rainfall (mm)
				Min	Max	Average	Difference	
Jinzhou	121°55’E	41°31’N	45	13.54	30.90	22.90	17.36	342.4
Fuxin	122°52’E	42°71’N	237	11.40	29.20	21.62	17.80	214.1
Weifang	119°15’E	36°77’N	17	16.61	32.80	25.13	16.19	501.1

**Table 3:** Comprehensive soil parameters for the experimental fields (Source: National Earth System Science Data Service Center, https://soil.geodata.cn/).

Location	Soil texture	Available K (ppm)	N (%)	Available P (ppm)	Organic carbon	pH
Jinzhou	Sandy loam	97	0.11	7	1.64	6.5
Fuxin	Sandy loam	99	0.03	4	0.55	7.2
Weifang	Sandy loam	127	0.65	9.9	1.12	6.7

### 2.2 Peanut roasting and preparation

The peanuts from each location were roasted in a T3-L383B Meidi oven (Meidi Ltd. Company, China). The oven temperature was maintained at 190°C for 5 and 7 minutes for roasting small-seeded and large-seeded genotypes, respectively. After roasting, all peanuts were allowed to cool down to room temperature in a natural way, then samples were packaged in food-grade plastic bags and stored in them, the sensory evaluation was carried out on the same day.

### 2.3 Sensory evaluation

An 8-member trained peanut profile panel (4 female and 4 male) at Shandong Peanut Research Institute, Qingdao, Shandong, participated in the spectrum descriptive analysis. All panelists were chosen based on the following specific criteria: (1) people who have experience in the evaluation of peanut products, (2) people are not allergic to peanuts, (3) people with natural dentition, (4) non-smokers, (5) non-drug users, (6) people who interested in participating, and (7) people who able to verbally communicate regarding the product (Nepote et al., 2009). Sensory panelists were trained and their performance assessed according to The Spectrum™ Method (Sensory Spectrum, Inc., Chatham, NJ, USA). All panelists selected for this study possessed over 1000 hours of experience in sensory evaluation and training specific to peanuts and peanut products. Their comprehensive training included terminology development, introduction to scaling, initial practice, discrimination of small product differences, and final calibration, all conducted using peanuts and peanut products as test materials. The panelists were given the attributes definitions (**Table 4**) on how to evaluate all samples using a five-point scale (e.g., 1=weak, 5=strong) for crunchiness (1 (weak) to 5 (strong)), fineness (1 (weak) to 5 (strong)), sweetness (1 (weak) to 5 (strong)), bitterness (1 (weak) to 5 (strong)), off-flavor (1(weak) to 5 (strong)), roasted peanutty (1(weak) to 5 (strong)) and overall acceptability (1 (dislike) to 5 (like)). Attributes definitions were developed based on relevant literature, previous research findings, and deliberations by the sensory panel (Johnsen et al., 1988; Nepote et al., 2006; Miyagi, 2017) (**Table 4**). The panelists evaluated samples from the same location at a time in a randomized complete block design with 3 replications. The order of samples was random for each panelist. Three seeds of each sample were tasted for texture evaluations and pasted samples were tasted for the flavor attributes, then recording the scores in the given form. Before testing each sample, panelists should rinse their mouths with warm water to minimize the influence of the previous sample. When testing with multiple panelists simultaneously, communication and conversation were not allowed.

**Table 4:** Definitions of attributes used by the trained panel to describe roasted peanuts.

Attribute	Definition
Texture	
1. Crunchiness	Force needed and amount of sound generated from chewing a sample with molar teeth
2. Fineness	Texture on fine or smooth of a sample after well chewed with molar teeth
Tastes	
3. Sweetness	Taste on the tongue associated with sucrose solutions
4. Bitterness	Taste on the tongue associated with bitter solutions such as quinine, caffeine, etc.
Aromatics	
5. Off-flavor	The aromatic associated with wet cardboard or rancid fats and oils
6. Roasted-peanutty	The aromatic associated with medium-roasted peanuts
Comprehensive assessment	
7. Overall acceptability	Assess preference for a sample by combining texture, tastes, and aroma characteristics

### 2.4 Analysis on biochemical quality of peanut kernels

Biochemical quality of peanut kernels inclusive of proanthocyanidin (Zhang, 2023), sucrose contents (Tang et al., 2018), oleic acid (Yu et al., 2010), linoleic acid (Yu et al., 2010), palmitic acid (Yu et al., 2010), oil (Yu et al., 2003), protein (Yu et al., 2003), and vitamin E (Liu et al., 2018) were analyzed by near infrared reflectance spectroscopy (NIRS) Matrix-I (Bruker Optics Inc., German), and the software version was OPUS 7.5. To reduce measurement errors, warm up the machine for at least 30 minutes. All samples were naturally sun-dried and were at room temperature for over 6 hours before scanning to minimize the impact of the material’s temperature on the results. Each sample was scanned 3 times and the average value was calculated.

### 2.5 Statistical analysis

The data were collected using Excel 2016 and analysis with DPS 18.1 software package. A multi-site randomized block design was used to evaluate the sensory attributes for the roasted peanuts, the biochemical components for the raw peanuts. A combined analysis of variance was first conducted according to the experiment design, with genotypes and locations as fixed effects. Variation in sensory attribute and biochemical component scores was partitioned into parts due to environment, genotype, G×E interaction, and error. Duncan’s multiple range test (DMRT) was utilized for multiple comparison. The GGE biplot analysis with Which-Won-Where, Mean versus Stability, Discrimitiveness versus representativeness and Ranking genotypes was done by using the R studio version 4.3.1 (Development Core Team, Vienna), the GGE Biplot GUI package. The relationship among crunchiness, fineness, sweetness, bitterness, off-flavor, roasted peanutty, and overall acceptability, and inter-relationships between peanut biochemical components of raw kernels, sensory attributes of roasted kernels were revealed by Pearson’s correlation analysis using R 4.3.1, the corrplot, ggplot2, and ggpubr packages.

## 3 Results

### 3.1 Variability in sensory attributes among genotypes and across locations

The results of the analysis of the variables and G × E interactions are presented in **Table 5**. All the sensory attributes exhibited significant differences at the 0.01 level due to genotype × location effects, and there were highly significant differences in the variability among the tested genotypes. Locations also had significant effects on all sensory attributes, except for off-flavor and roasted peanutty. In other words, there was no significant difference in the aroma attribute among locations after roasting. There were no significant differences in the variability among blocks in locations for all sensory attributes.

**Table 5:** Mean squares from analyses of variance for the sensory quality of roasted peanuts.

Source of variation	DF	Crunchiness		Fineness		Sweetness		Bitterness		Off-flavor		Roasted Peanutty		Overall acceptability	
		MS	TVP (%)	MS	TVP (%)	MS	TVP (%)	MS	TVP (%)	MS	TVP (%)	MS	TVP (%)	MS	TVP (%)
block in locations	6	0.0060	0.38	0.0538	1.26	0.0288	0.93	0.0226	2.55	0.0187	1.85	0.0463	1.36	0.0051	0.16
locations	2	0.1959^**^	4.13	1.6396^**^	14.59	0.9503^**^	10.21	0.1223^**^	4.60	0.0365	12.04	0.0893	3.85	1.9280^**^	20.37
genotype	12	0.3450^**^	43.65	0.9792^**^	45.99	0.8098^**^	60.01	0.0919^**^	20.75	0.1435^**^	17.70	0.4504^**^	25.18	0.4692^**^	29.80
genotype× location	24	0.1351^**^	34.20	0.3641^**^	34.20	0.1514^**^	19.56	0.1047^**^	47.24	0.1143^**^	45.29	0.4996^**^	58.37	0.2624^**^	33.31
error	72	0.0232	17.64	0.0203	5.71	0.0240	9.30	0.0184	24.87	0.0203	23.13	0.0309	10.87	0.0430	16.37

**was presented significant differences p < 0.01. DF stands for degrees of freedom. MS and TVP are the abbreviation of mean square and total variance proportion, respectively.

As shown in **Table 6**, peanuts from Weifang showed great crunchiness, more fineness, high sweetness and low bitterness compared to those from the other two locations. Regarding the aroma attribute, there was no significant difference in off-flavor and roasted peanutty among the three locations. Peanuts from Weifang obtained the highest score in overall acceptability.

**Table 6:** The evaluation results of sensory attributes among locations.

Locations	Crunchiness	Fineness	Sweetness	Bitterness	Off-flavor	Roasted peanutty	Overall acceptability
Jinzhou	3.70b	3.70b	3.43b	1.25b	1.25a	3.88ab	3.00b
Fuxin	3.73b	3.60c	3.34c	1.33a	1.31a	3.87ab	3.10b
Weifang	3.88a	3.99a	3.65a	1.22b	1.24a	3.96a	3.43a

Significant differences (p < 0.05) were determined using Duncan’s multiple range test. Means with different letters within the same row are significantly different.

The means for the 13 genotypes tested (**Table 7**) indicated that the ranges of crunchiness, fineness, sweetness, bitterness, off-flavor, roasted peanutty, and overall acceptability. The ranges of the fineness and roasted peanutty attributes for small-seeded and large-seeded peanuts were overlapping, and the small-seeded types showed a significantly (P ≤0.05) higher mean than the large-seeded types for fineness (4.02 vs. 3.59 intensity units), and a significantly (P ≤ 0.05) lower mean for roasted peanutty (3.78 vs. 3.98 intensity units). Among all genotypes, S9 had the best sensory attributes, with high crunchiness and sweetness, and at the same time, low bitterness and off-flavor. L10 had higher crunchiness, high roasted peanutty, and the highest overall acceptability among the large-seeded peanuts.

**Table 7:** Mean sensory attribute scores for roasted peanut genotypes.

Genotypes	Crunchiness	Fineness	Sweetness	Bitterness	Off-flavor	Roasted peanutty	Overall acceptability
S1	3.54fg	3.93c	3.67bc	1.11e	1.19cde	3.81d	2.94de
S11	3.60fg	3.63d	3.18e	1.24bcde	1.35bc	4.11ab	3.21c
S25	3.94bc	3.89c	3.51cd	1.32abc	1.31bc	3.51d	2.86e
S9	4.10a	4.51a	4.19a	1.10e	1.07e	3.88c	3.67a
Huayu20	3.81cde	4.18b	3.08e	1.26bcd	1.21cde	3.60d	3.19c
L1	3.69ef	3.64d	3.71b	1.38ab	1.53a	3.97bc	3.18c
L10	4.00ab	3.57d	3.59bcd	1.17de	1.22cd	4.19a	3.47ab
L11	3.83cde	3.64d	3.10e	1.32abc	1.39ab	4.06ab	3.15c
L15	3.68ef	3.54d	3.48d	1.26bcd	1.31bc	3.94bcd	3.11cd
L17	3.78de	3.29e	3.56bcd	1.32abc	1.21cde	4.17a	3.32bc
L8	3.50g	3.38e	3.71b	1.19cde	1.11de	4.07ab	3.17c
L9	3.86bcd	3.86c	3.18e	1.43a	1.42ab	3.83c	3.17c
Huayu33	3.49g	3.83c	3.22e	1.35ab	1.28bc	3.61d	2.83e

Significant differences (p < 0.05) were determined using Duncan’s multiple range test. Means with different letters within the same row are significantly different.

**Figure 1:**
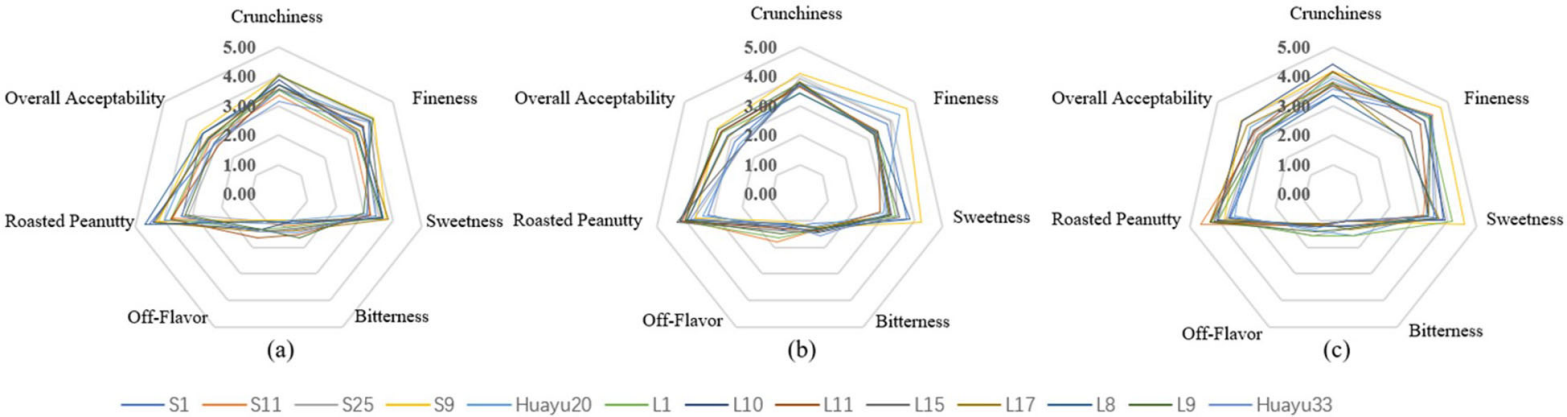
The sesory attribute scores of 13 genotypes for roasted peanuts in 3 locations: **(a–c)** – The sensory attribute scores of 13 roasted peanuts in Jianzhou, Fuxin and Weifang respectively.

From **Figure 1**, there is a visual presentation of the differences in sensory attributes among the three different locations for roasted peanuts.

### 3.2 Genotype and genotype by environment for sensory attributes

**Figure 2:**
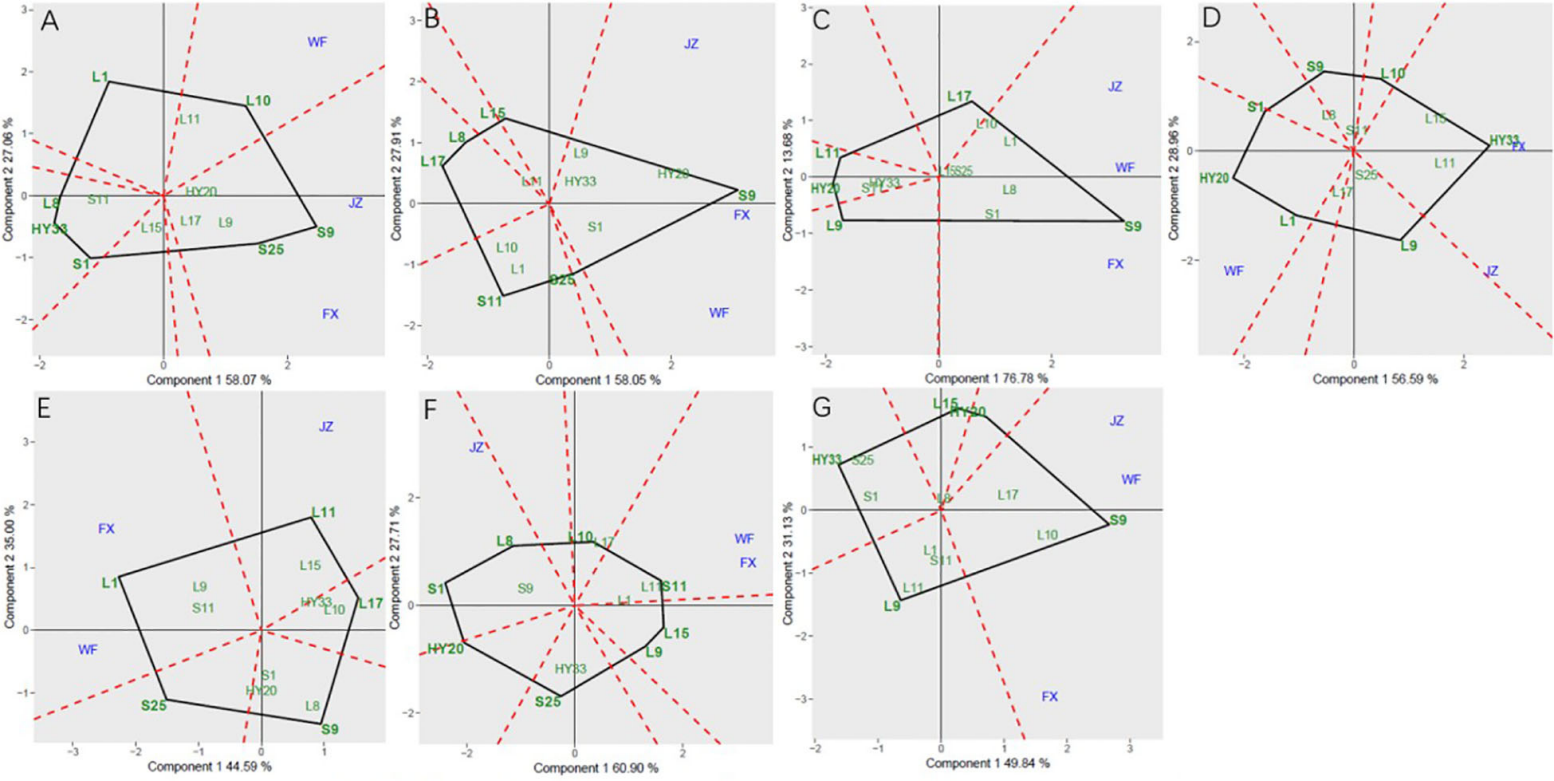
Polygon views of GGE biplot for which-won-where analysis of 13 genotypes under 3 environments. **(A)** crunchiness, **(B)** fineness, **(C)** sweetness, **(D)** bitterness, **(E)** off-flavor, **(F)** roasted peanut, **(G)** overall acceptability. The GGE biplot polygons were based on scaling = 1, and singular value partitioning (SVP) = 3.

**Figure 3:**
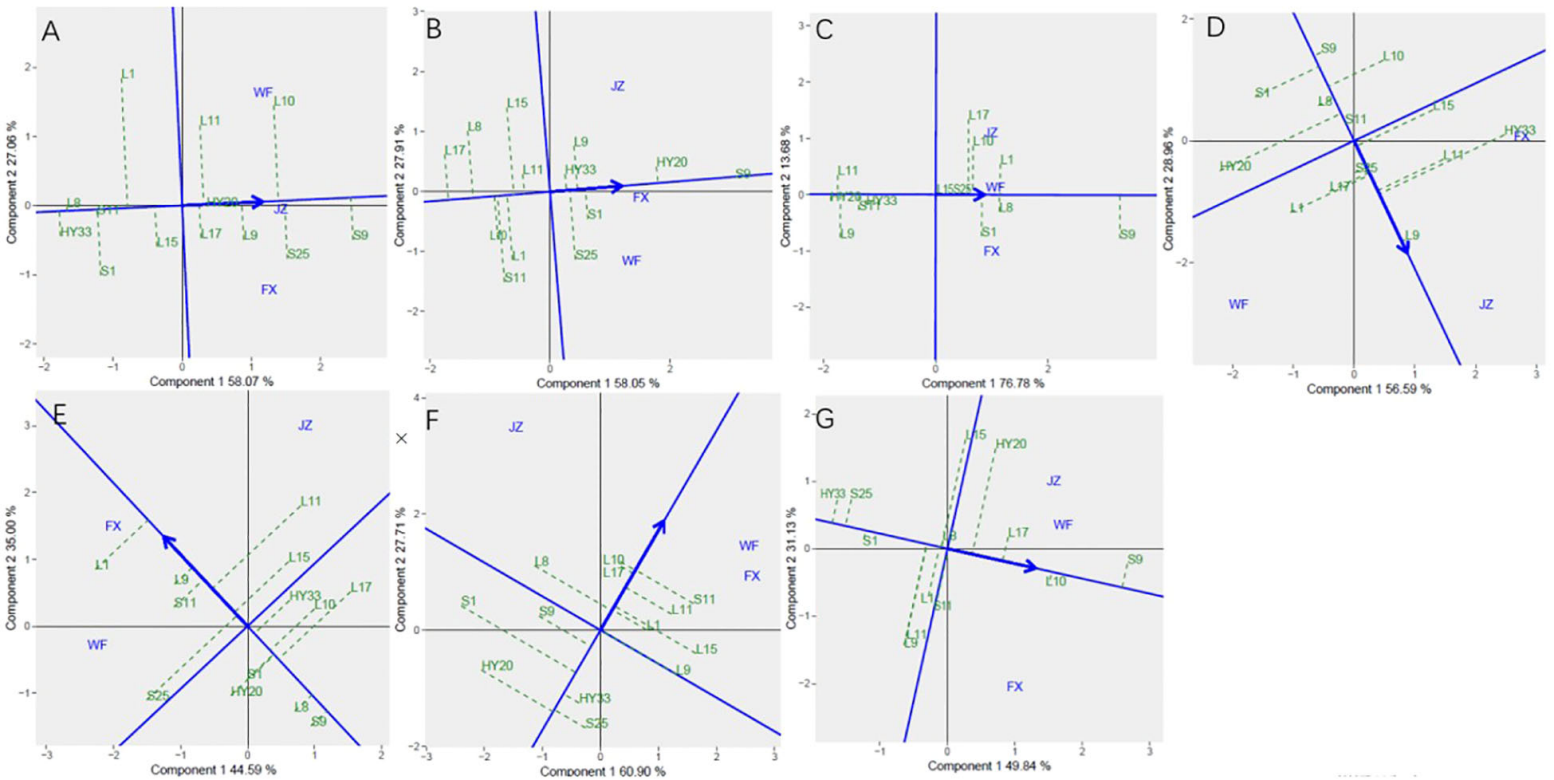
Mean vs. stability based on Component 1 and Component 2 showing GXE interactions of 13 genotypes under 3 environments. **(A)** crunchiness, **(B)** fineness, **(C)** sweetness, **(D)** bitterness, **(E)** off-flavor, **(F)** roasted peanutty, **(G)** overall acceptability. The mean vs. stability was based on scaling =1, centering = 2, and singular value partitioning (SVP) =1.

The GGE biplot for which-won-where of sensory attributes (**Figures 2A–G**) showed that the Component 1 and Component 2 accounted for 85.13%, 85.96%, 90.46%, 85.55%, 79.59%, 88.61%, and 80.97% of total variation for crunchiness, fineness, sweetness, bitterness, off-flavor, roasted peanutty, and overall acceptability, respectively (**Table 8**). The polygon containing all genotypes was formed by lines which connecting the signs of the genotypes that are farthest away from the biplot origin, and then draw the perpendicular lines to each side of the polygon from origin point, which divided the whole biplot into several sectors. Sectors help to visualize the mega-environments. The winning genotypes for each sector are placed at the vertex. The test environments fell into two of the seven sectors, one of the six sectors, one of the five sectors, two of the seven sectors, two of the five sectors, two of the eight sectors, and one of the five sectors outlined on the polygon view for crunchiness, fineness, sweetness, bitterness, off-flavor, roasted peanutty, and overall acceptability, respectively (**Figure 2**). Thus, the mega-environments were identified for each sensory attribute. There were one mega-environment for fineness, sweetness, and overall acceptability, respectively. There were two mega-environments for crunchiness and bitterness with FX and JZ grouped together in a mega-environment, while WF is in the second mega-environment. There were two mega-environments for off-flavor and roasted peanutty with WF and FX grouped together in a mega-environment, while JZ is in the second mega-environment. From **Figure 2A**, 3 locations were divided into 2 sectors, S9 was the best performer at FX and JZ, and L10 was the vertex genotype at WF in crunchiness. From **Figures 2B, C, G**, 3 locations were belonged to 1 sector, S9 was the vertex genotype at this environment in fineness, sweetness, and overall acceptability, respectively.

**Table 8:** Principal component analysis of sensory attributes.

Sensory attributes	PC1/%	PC2/%	GGE variation (%)
Crunchiness	58.07	27.06	85.13
Fineness	58.05	27.91	85.96
Sweetness	76.78	13.68	90.46
Bitterness	56.59	28.96	85.55
Off-flavor	44.59	35.00	79.59
Roasted Peanutty	60.90	27.71	88.61
Overall Acceptability	49.84	31.13	80.97

The **Figure 3** showed the “mean vs. stability” pattern of GGE biplot for sensory attributes of the 13 peanut genotypes evaluated. The abscissa and the ordinate of the average environment coordinate (AEC) axis are the two lines passing through the origin of the biplot, and the AEC showed the trend of average sensory attribute scores of peanuts across different locations. The abscissa points toward increased order of genotype performance. A perpendicular line drawn from the origin to the AEC revealed the tendency of genotype-environment interaction (GEI). The projection on the abscissa toward the ordinate of the AEC is a measure of stability, so a genotype with zero projection or very short direction from the ordinate is considered the most stable, while a genotype with the longest projection from the abscissa is unstable. In our study, the ordinate divides the genotypes into two: those that sensory attributes above and below average. **Figure 3A** showed the genotypes toward the arrow that crunchiness above the average means: S9, S25, L10, L9, HY20, L11, and L17; In contrast, the following fell below the average means: L15, L1, S11, S1, L8, and HY33. Based on crunchiness, fineness, sweetness, bitterness, off-flavor, roasted peanutty, and overall acceptability, S9, S9, S9, L9, L1, L10 and S9 were the highest genotypes, while HY33, L17, HY20, S9, S9, HY20, and HY33 were the lowest genotypes, and HY20, S9, HY20, S25, L9, L17, and S1 had the shortest projection, while L1, S11, L17, HY33, S25, S1, and L15 had the longest.

**Figure 4:**
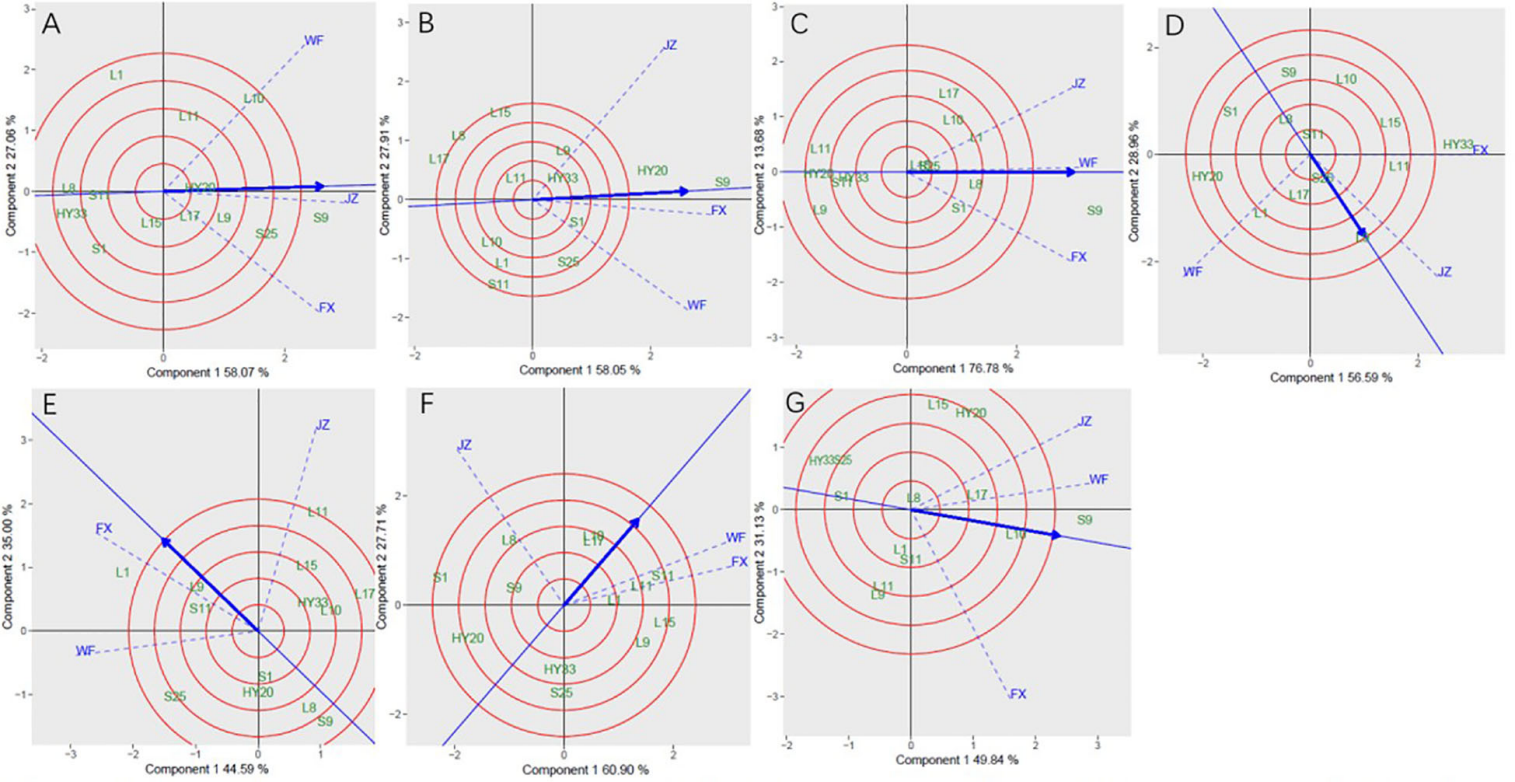
Discrimitiveness vs. representativenss GGE biplot for sensory attributes of 13 genotypes under 3 environments. **(A)** crunchiness, **(B)** fineness, **(C)** sweetness, **(D)** bitterness, **(E)** off-flavor, **(F)** roasted peanutty, **(G)** overall acceptability. The discrimitiveness vs. representativenss were based on scaling = 1. centering = 2, and singular value partitioning (SVP) = 3.

Discriminativeness vs. representativeness pattern of the GGE biplot for sensory attributes was presented in **Figure 4**. They pinpoint how the best environment can be informative and representative. The two concepts focus on environments in terms of their ability to detect the best genotypes (discriminativeness) and to adequately represent the test environments (representativeness) (Yan and Tinker, 2006; Oladosu et al., 2017; Khan et al, 2021). The vector length for each environment revealed the discriminatory ability of the environment, while the angle formed by each vector with the abscissa denotes representativeness. The longer the vector of an environment, the higher its capability to discriminate among genotypes, while the shorter the angle formed with the abscissa, the more it is representative. The shortest vector for Patterns B, D, and F was FX, for Patterns C, E, and G was FX, and for Pattern A was JZ, while the longest vector for Patterns A and B was WF, for Patterns C, D, E and F was JZ, and for Patterns G was FX. The angles the environment formed with the abscissa line were also recorded. For crunchiness, the shortest angle was formed by JZ. For fineness, bitterness, and roasted peanutty the shortest angle was formed by FX. For sweetness, off-flavor, and overall acceptability, the shortest angle was formed by WF. The longest angles formed by the environment were also observed to be FX for sweetness and overall acceptability, and JZ for fineness, off-flavor, and roasted peanutty, while for crunchiness and bitterness, they were formed by WF. The biplot identified the environments that were closest to the AEC. The environments were JZ for crunchiness, and FX for fineness, bitterness, and roasted peanutty, while WF for sweetness, off-flavor, and overall acceptability.

**Figure 5:**
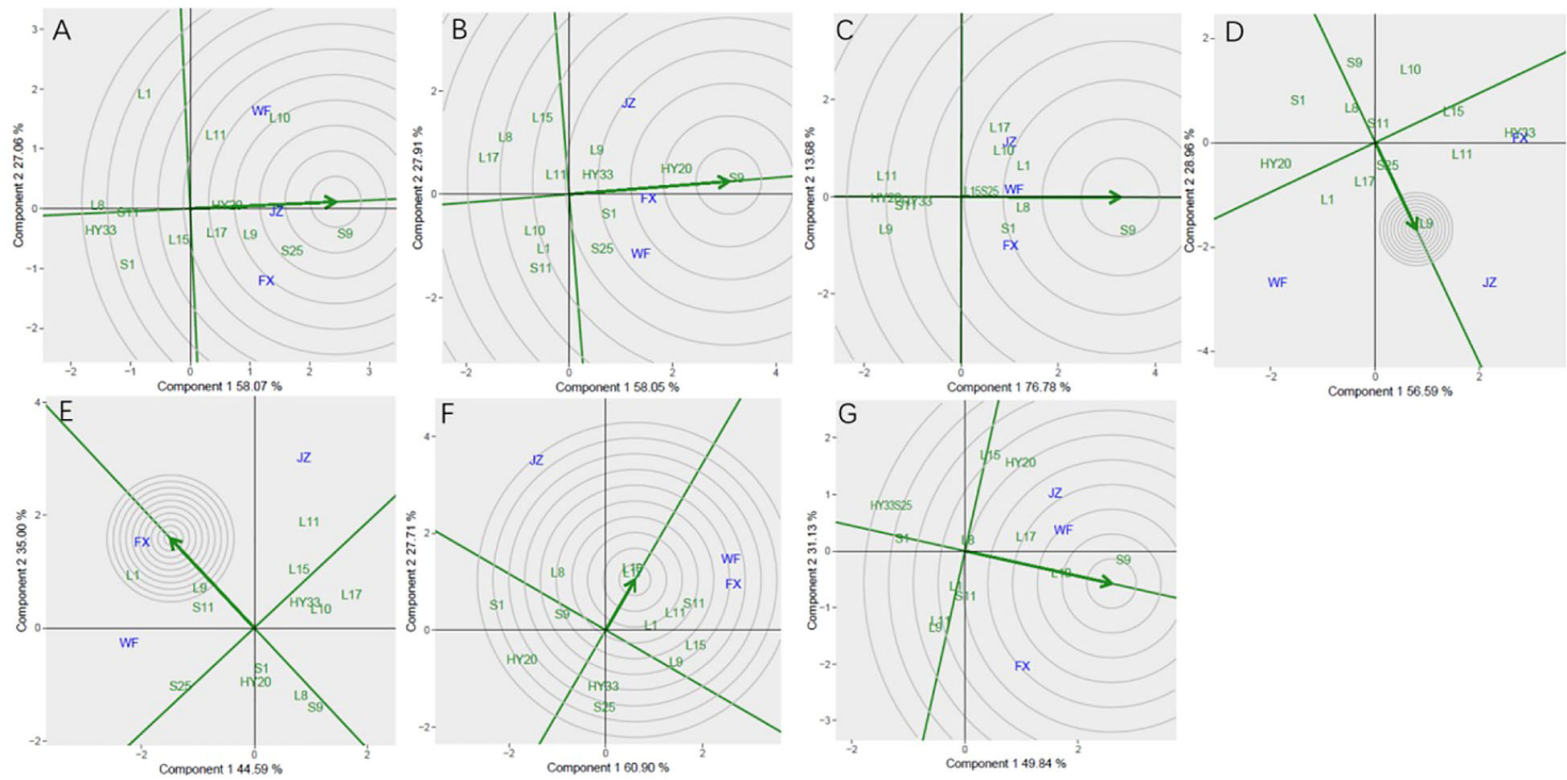
Ranking genotypes of sensory attributes of 13 genotypes under 3 environments. **(A)** crunchiness, **(B)** fineness, **(C)** sweetness, **(D)** bitterness, **(E)** off-flavor, **(F)** roasted peanutty, **(G)** overall acceptability. The ranking genotypes were based on scaling 1, centering = 2, and singular value partitioning (SVP) = 1.

In **Figure 5**, genotype ranking based on PC1 and PC2 of sensory attributes showing G×E interactions of 13 peanut genotypes under 3 locations. The biplot allowed us to identify the best and ideal genotype among the 13 tested genotypes. An ideal genotype is always localized into the innermost circle and virtually closer to the head of the arrow at the center of the circular ring (Esan et al., 2023) (**Figure 5**: Patterns A, B, C, D, E, F and G). The only genotype that fell inside the inner circle was S9; the genotypes next to the ideal inner circle were S25 and L10, followed by L9, HY20, L17, and L11 for crunchiness (**Figure 5A**). Similarly, for fineness, S9, HY20 and S1 were next to the ideal genotype (**Figure 5B**). For sweetness, the only genotype was S9, while L8, L1, S1, L10, S25, L15 and L17 followed the only ideal genotype (**Figure 5C**). For roasted peanutty, the ideal inner circle was L17 and L10 (**Figure 5F**). For overall acceptability, the ideal inner circle was S9, followed by L10 and L17 (**Figure 5G**).

### 3.3 Variability in biochemical components among genotypes and across locations

The results of the analysis of the variable components and G×E interactions are listed in **Table 9**. All the biochemistry components exhibited significant differences at the 0.05 or 0.01 level due to the effects of locations, genotypes, and genotype × location. There were no significant differences in the variability among blocks in locations except for oil.

**Table 9:** Mean squares from analyses of variance for biochemical components of raw peanuts.

Source of variation	DF	Proanthocyanidin		Sucrose		Oleic		Linoleic		Palmitic		Oil		Protein		Vitamin E	
		MS	TVP (%)	MS	TVP (%)	MS	TVP (%)	MS	TVP (%)	MS	TVP (%)	MS	TVP (%)	MS	TVP (%)	MS	TVP (%)
block in locations	6	0.0008	0.11	0.0491	0.70	0.4512	0.21	0.1107	0.0046	0.0410	0.06	0.4228^*^	1.06	0.1982	1.22	0.2157	1.02
locations	2	0.1016^**^	4.31	9.3775^**^	44.76	1.6457^*^	0.24	3.8249^**^	0.0485	1.5093^**^	0.71	7.4365^**^	6.21	13.4300^**^	27.54	5.4079^**^	8.50
genotype	12	0.3050^**^	77.76	1.6090^**^	46.09	1373.8379^**^	99.50	1206.5486^**^	99.69	34.1687^**^	96.95	13.5949^**^	68.12	2.9956^**^	36.85	6.7561^**^	63.62
Genotype ×location	24	0.0299^**^	15.24	0.0654^**^	3.75	1.3531^**^	0.25	0.9690^**^	0.16	0.26710^**^	1.23	1.5707^**^	15.74	0.6524^**^	16.05	1.2053^**^	19.31
error	72	0.0017	2.58	0.0273	4.70	0.3748	0.21	0.1896	0.094	0.0335	1.04	0.2953	8.85	0.2485	18.34	0.1342	7.57

*and ** was presented significant differences p < 0.05 and p < 0.01, respectively. DF stands for degrees of freedom. MS and TVP are the abbreviation of mean square and total variance proportion, respectively.

As shown in **Table 10**, the content of proanthocyanidin and vitamin E in peanuts from Weifang had a significantly (P ≤ 0.05) lower mean, while the content of sucrose and protein in peanuts from Weifang had a significantly (P ≤ 0.05) higher mean than that of the peanuts from the other two locations. The content of vitamin E in the peanuts from the Northeast peanut-growing area in China (Jinzhou and Fuxin) was significantly higher than that from the Shandong peanut-growing area.

**Table 10:** The evaluation results of biochemical components among locations.

Location	Proanthocyanidin	Sucrose	Oleic	Linoleic	Palmitic	Oil	Protein	Vitamin E
Jinzhou	1.80b	4.94c	72.01b	10.77a	7.13b	52.18a	26.15c	12.08a
Fuxin	1.82a	5.15b	72.33a	10.23b	7.46a	51.52b	26.51b	12.10a
Weifang	1.73c	5.88a	72.40a	10.23b	7.10b	51.35b	27.29a	11.45b

Significant differences (p < 0.05) were determined using Duncan’s multiple range test. Means with different letters within the same row are significantly different.

The means for the 13 genotypes tested (**Table 11**) showed the ranges of proanthocyanidin, sucrose, oleic, linoleic, palmitic, oil, protein and vitamin E for raw peanuts. There was no significant difference in the biochemical components of peanuts between the large-seeded and small-seeded peanuts, except for proanthocyanidins. The mean content of proanthocyanidin of large peanuts (1.89 mg/g) was significantly higher (P ≤ 0.05) than that of small peanuts (1.62 mg/g). The three small-seeded genotypes S1, S9 and Huayu20 had the low content of proanthocyanidin compared with the other genotypes.

**Table 11:** Mean content of biochemical components of 13 raw peanuts.

Genotypes	Proanthocyanidin (mg/g)	Sucrose (g/100g)	Oleic Acid (%)	Linoleic Acid (%)	Palmitic Acid (%)	Oil (%)	Protein (g/100g)	Vitamin E (mg/100g)
S1	1.52f	4.90e	77.15c	6.52c	6.80f	52.32c	26.23de	13.13a
S11	1.72e	5.56bc	78.59b	4.26e	6.07g	50.86def	27.21ab	9.90e
S25	1.78d	5.44c	80.38a	3.21f	5.47i	50.49f	26.44cde	13.14a
S9	1.54f	5.98a	80.08a	4.16e	5.82h	52.35c	26.75bcd	11.19d
Huayu20	1.52f	4.27f	44.75f	36.39a	11.12b	54.47a	27.23ab	12.53b
L1	2.02a	5.47c	75.77d	6.72c	7.50c	51.09de	26.26de	11.81c
L10	1.78d	5.52bc	76.20d	6.87c	7.31d	51.06de	27.13ab	12.59b
L11	2.03a	5.41c	77.37c	3.95e	5.59i	51.21d	26.21e	11.77c
L15	1.93b	5.43c	76.90c	4.76d	5.49i	51.09de	27.59a	11.80c
L17	1.80cd	5.68b	75.719d	7.98b	7.12e	50.50f	26.91bc	11.29d
L8	1.83c	5.44c	75.73d	7.69b	7.04e	50.58ef	26.75bcd	11.50cd
L9	1.70e	5.13d	76.07d	6.69c	7.20de	53.35b	26.28de	11.88c
Huayu33	2.01a	5.00de	45.54e	36.16a	11.47a	52.52c	25.45f	11.87c

Significant differences (p < 0.05) were determined using Duncan’s multiple range test. Means with different letters within the same row are significantly different.

### 3.4 Genotype and genotype by environmen for biochemical components

**Figure 6:**
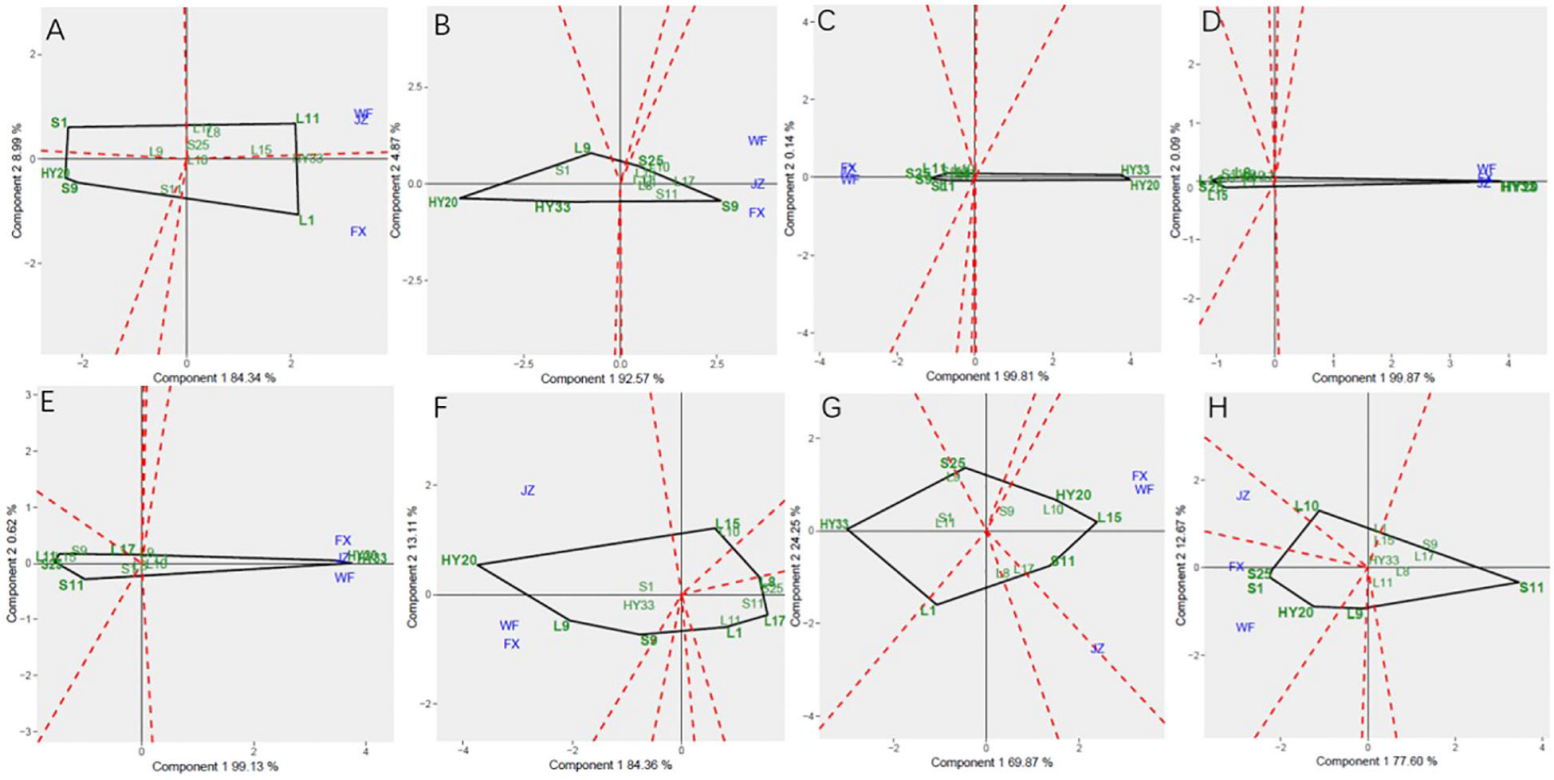
Polygon views of GGE biplot for which-won-where analysis of 13 genotypes under 3 environments. **(A)** proanthocyanidins, **(B)** sucrose, **(C)** oleic acid, **(D)** linoleic acid, **(E)** palmitic acid, **(F)** oil, **(G)** protein, **(H)** vitamin **(E)** The GGE biplot polygons were based on scaling = 1, centering = 2, and singular value partitioning (SVP) = 3.

The GGE biplot for which-won-where of biochemical components (**Figures 6A–H**) showed that the Component 1 and Component 2 accounted for 93.33%, 92.57%, 99.95%, 99.96%, 99.75%, 97.47%, 94.12%, and 90.27% of total variation for proanthocyanidin, sucrose, oleic, linoleic, palmitic, oil, protein and vitamin E, respectively (**Table 12**). The test environments fell into two of the five sectors, one of the five sectors, one of the seven sectors, one of the six sectors, one of the six sectors, one of the six sectors, one of the seven sectors, two of the six sectors, and two of the six sectors outlined on the polygon view for proanthocyanidin, sucrose, oleic, linoleic, palmitic, oil, protein and vitamin E, respectively (**Figure 6**). Thus, the mega-environments were identified for each biochemical component. There were one mega-environment for sucrose, oleic, linoleic, palmitic, and oil, respectively. There were two mega-environments for proanthocyanidin with WF and JZ grouped together in a mega-environment, while FX is in the second mega-environment. There were two mega-environments for protein and vitamin E with WF and FX grouped together in a mega-environment, while JZ is in the second mega-environment. From **Figure 6A**, 3 locations were divided into 2 sectors, one environment was WF and JZ, and FX was another one. L11 was the best performer at JZ and WF, and L1 was the vertex genotype at FX for proanthocyanidin. From **Figures 6G, H**, 3 locations were divided into 2 sectors, one environment was WF and FX, and JZ was another one. L15 was the best performer at FX and WF, and L17 was the vertex genotype at JZ for protein (**Figure 6G**), while S1 was the best performer at FX and WF, and no vertex genotype at JZ for Vitamin E (**Figure 6H**). From 6B-6F, 3 locations were belonged to 1 sector, S9, S25, HY20, HY33, and HY20 was the vertex genotype at this environment in sucrose, oleic acid, linoleic acid, palmitic acid, and oil, respectively.

**Table 12:** Principal component analysis of biochemical components.

Sensory attributes	PC1/%	PC2/%	GGE variation (%)
Proanthocyanidin	84.34	8.99	93.33
Sucrose	92.57	4.87	92.57
Oleic Acid	99.81	0.14	99.95
Linoleic Acid	99.87	0.09	99.96
Palmitic Acid	99.13	0.62	99.75
Oil	84.36	13.11	97.47
Protein	69.87	24.25	94.12
Vitamin E	77.60	12.67	90.27

**Figure 7:**
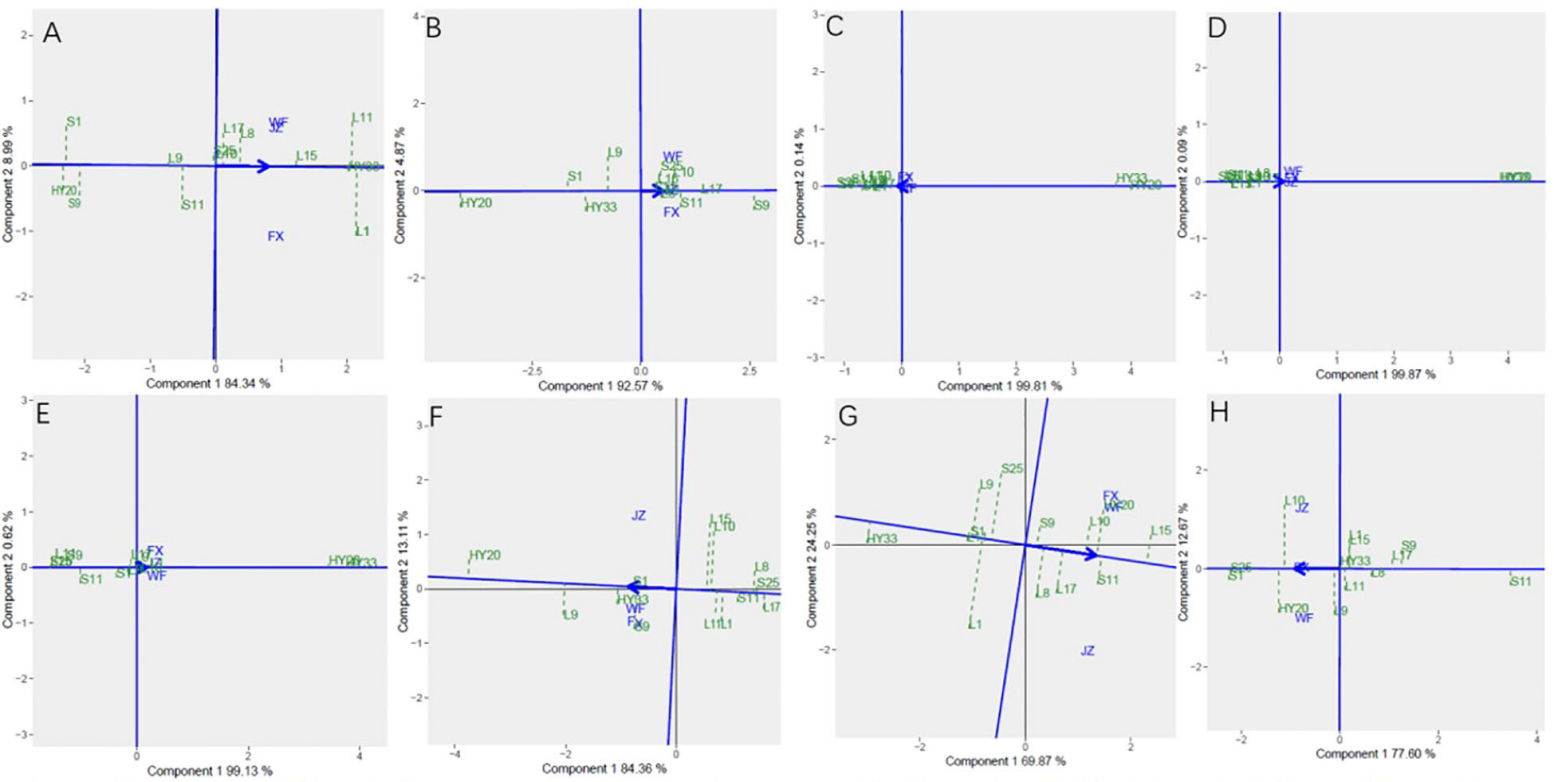
Mean vs. stability based on component 1 and component 2 showing GXE interactions of 13 genotypes under 3 environments. **(A)** proanthocyanidins, **(B)** sucrose, **(C)** oleic acid, **(D)** linoleic acid, **(E)** palmitic acid, **(F)** oil, **(G)** protein, **(H)** vitamin **(E)** The mean vs. stability was based on scaling =1, centering = 2, and singular value partitioning (SVP) =1.

The **Figure 7** showed the “Mean vs. stability” pattern of GGE biplot for biochemical components of the 13 peanut genotypes evaluated. Based on proanthocyanidin, sucrose, oleic, linoleic, palmitic, oil, protein and vitamin E, L1, S9, S25, HY20, HY33, HY20, L15, and S25 were the highest genotypes, while HY20 (S1), HY20, HY20, S25, S25, L17, HY33, and S11 were the lowest genotypes. For proanthocyanidin, sucrose, oil, protein and vitamin E, L1, L9, L15, L1, and L10 had the longest projection, while HY33, L17, S1, L11, and S25 had the shortest. For oleic, linoleic and palmitic acid, we couldn’t identify the length of the projection through the **Figures 7C–E**.

**Figure 8:**
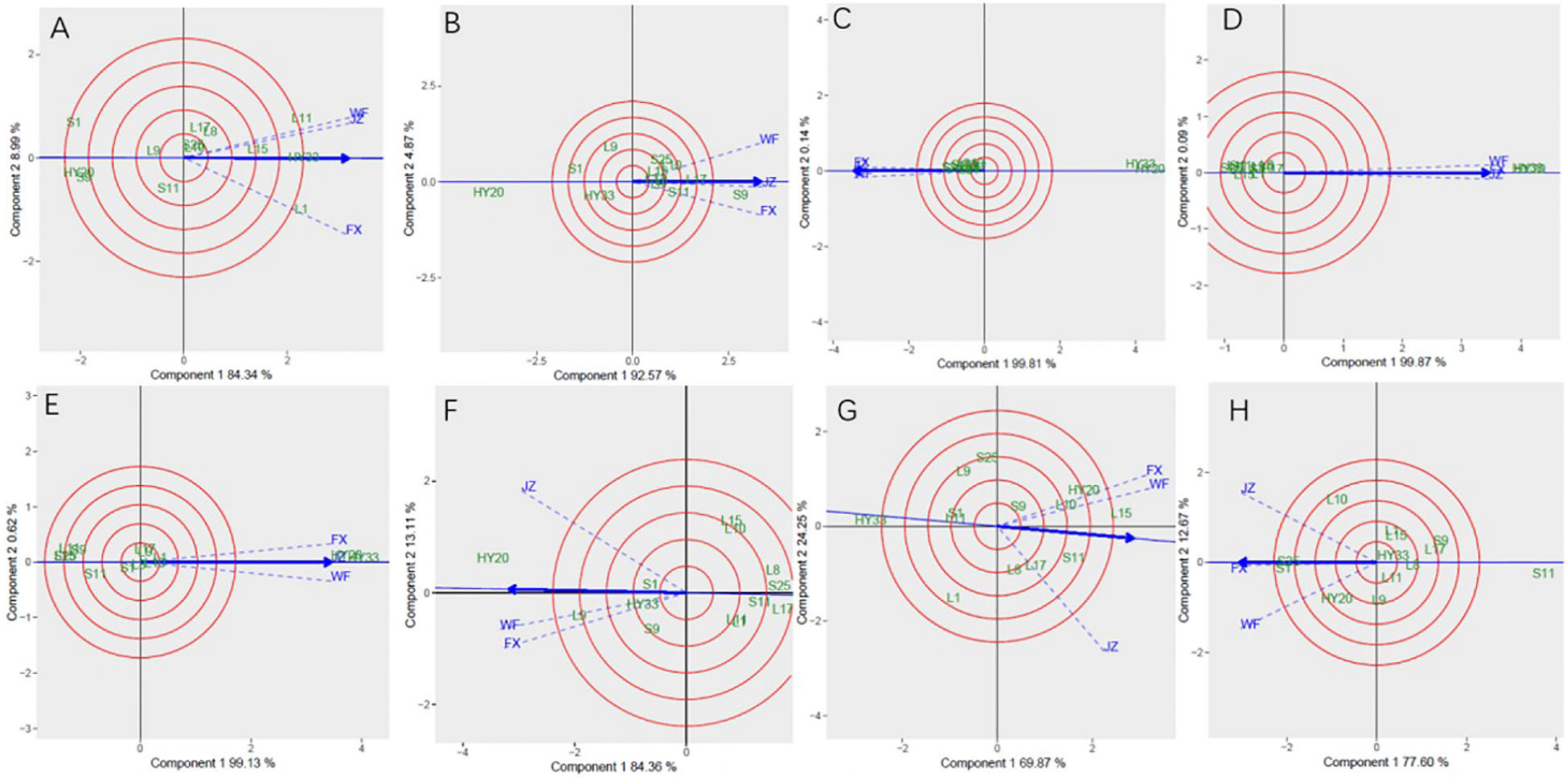
Discrimitiveness vs. representativenss GGE biplot of 13 genotypes under 3 environments. **(A)** proanthocyanidins, **(B)** sucrose, **(C)** oleic acid, **(D)** linoleic acid, **(E)** palmitic acid, **(F)** oil, **(G)** protein, **(H)** vitamin **(E)** The discrimitiveness vs. representativenss were based on scaling = 1, centering = 2, and singular value partitioning (SVP) = 3.

Discriminativeness vs. representativeness pattern of the GGE biplot for biochemical components was shown in **Figure 8**. The shortest vector for Patterns A, B, C, E, and G was JZ and for Patterns D, F, and H was FX, while the longest vector for Patterns A and C was FX and for Patterns B, E, and G was WF, and Patterns D, F and H was JZ. The angles the environment formed with the abscissa line were also recorded. For proanthocyanidin, sucrose, oleic, and palmitic, the shortest angle was formed by JZ. For oil and protein, the shortest angle was formed by WF. For linoleic and vitamin E, the shortest angle was formed by FX. The longest angles formed by the environment were also observed to be FX for proanthocyanidin, and JZ for oil, protein and vitamin E, while for sucrose and three fatty acids, they were formed by WF. The biplot identified the environments that were closest to the AEC. The environments were WF for oil and protein, while JZ for proanthocyanidin, sucrose, oleic, and palmitic, and FX for linoleic and vitamin E.

**Figure 9:**
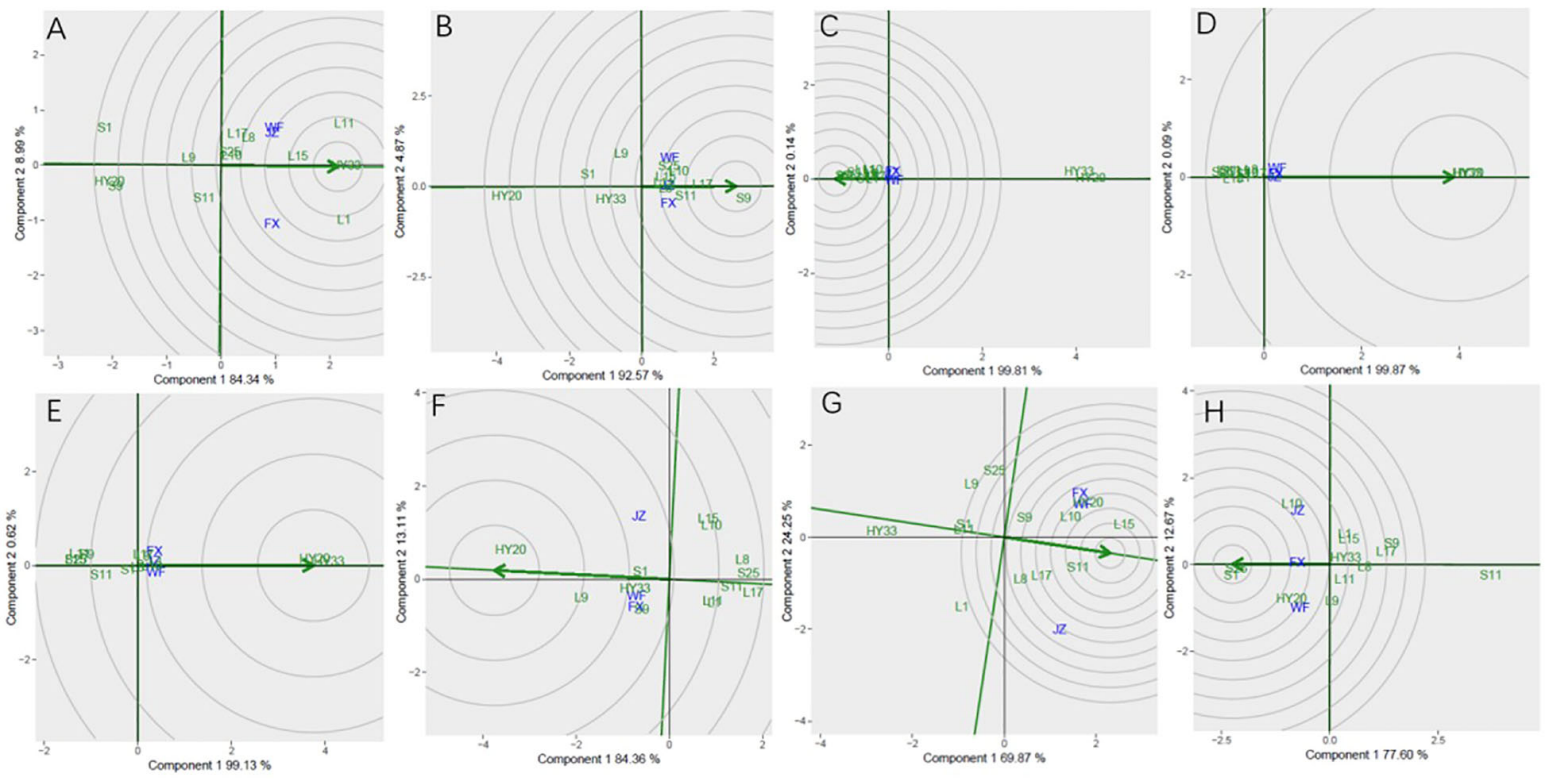
Ranking genotypes of biochemical components of 13 genotypes under 3 environments. **(A)** proanthocyanidins, **(B)** sucrose, **(C)** oleic acid, **(D)** linoleic acid, **(E)** palmitic acid, **(F)** oil, **(G)** protein, **(H)** vitamin **(E)** The ranking genotypes were based on scaling = 1, centering = 2, and singular value partitioning (SVP) = 1.

In **Figure 9**, genotype ranking based on PC1 and PC2 of biochemical components showing G×E interactions of 13 peanut genotypes under 3 locations. The only genotype that fell inside the inner circle was HY33, followed by L11, L15 and L1 for proanthocyanidin (**Figure 9A**). The only genotype that fell inside the inner circle was S9, followed by L17 and S11 for sucrose (**Figure 9B**) and the best genotype HY20 for oil (**Figure 9F**). For oleic, the genotypes except HY20 and HY33 were all inside the circle (**Figure 9C**). For linoleic and palmitic, HY20 and HY33 fell inside the inner circle (**Figures 9D, E**). The genotypes next to the ideal inner circle were S11 and L15, followed by HY20, L10, L17, L8, and S9 for protein (**Figure 9G**). For vitamin E, the genotypes S1 and S25 were all inside the inner circle (**Figure 9H**).

### 3.5 Correlation analysis in sensory attributes and biochemical components

**Figure 10:**
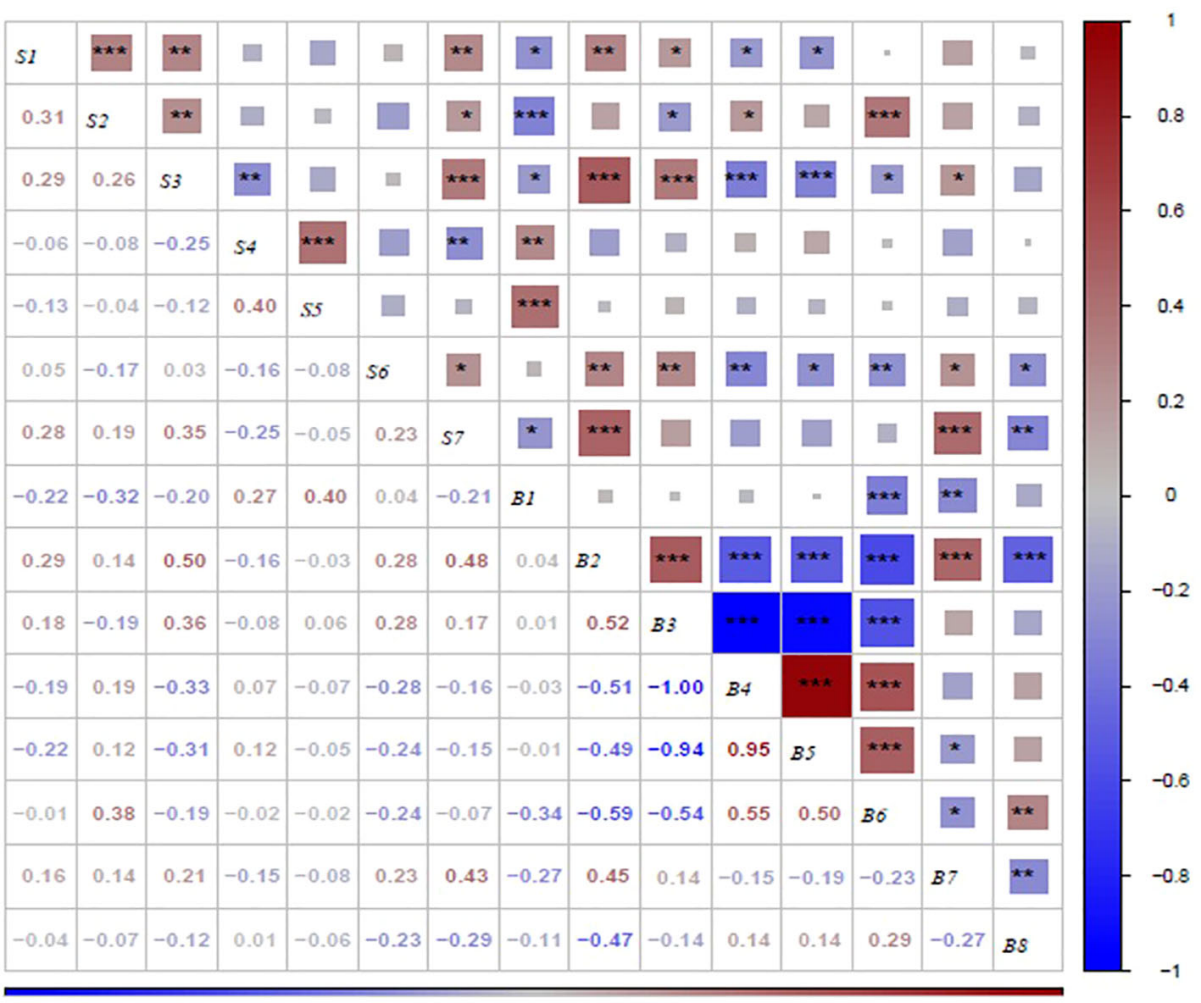
Correlation analysis between biochemical components and sensory attributes on roasted peanut. *,** and *** indicate difference significance at the P< 0.005, P<0.001 and P<0.001, respectively. S1, S2, S3, S4, S5, S6 and S7 were crunchiness, fineness, sweetness, bitterness, off-flavor, roasted peanutty, and overall acceptability, respectively. B1, B2, B3, B4, B5, B6, B7 and B8 were proanthocyanidins, sucrose, oleic acid, linoleic acid, palmitic acid, oil, protein, and vitamin E, respectively.

Correlation analysis was used to determine the relationship among all the sensory attributes in roasted peanuts (**Figure 10**). Overall acceptability was significantly positively correlated with crunchiness (r=0.28), fineness (r=0.19), sweetness (r=0.35), roasted peanutty(r=0.23) and significantly negatively correlated with bitterness(r=-0.25). Sweet-ness was significantly positively correlated with fineness (r=0.26), and significantly negatively correlated with bitterness (r=-0.25). Crunchiness was highly positive significant with fineness, sweetness and overall acceptability and highly negative significant with off-flavor.

The results presented in **Figure 10** demonstrate the correlations between sensory attributes and biochemical components in this study. Proanthocyanidins exhibited a highly significant negative correlation with crunchiness, fineness, sweetness and overall acceptability, while showing a highly significant positive correlation with bitterness and off-flavor. Sucrose demonstrated a highly significant positive correlation with sweetness and a positive correlation with crunchiness. The fatty acid content was found to be correlated with crunchiness, fineness, sweetness, roasted peanutty and overall acceptability. Oil was negatively correlated with sweetness and roasted peanutty. Protein was negatively correlated with bitterness, and positively correlated with sweetness and roasted peanutty. Vitamin E was negatively correlated with roasted peanutty. In terms of overall acceptability, it was negatively correlated with Vitamin E and proanthocyanidin, and positively correlated with sucrose, oleic acid, and protein.

## 4 Discussion

Previous studies have investigated the correlation between genotype and environment of the peanut sensory attributes, focusing solely on sweetness, bitterness, astringency, fruity, and roasted peanuts (Isleib et al., 2008b; Pattee et al., 1994, 1995, 1997, 1998). There are scanty reports of G × E interaction on sensory attributes for roasted peanuts in Chinese. Sensory attributes related studies have concentrated on the organoleptic evaluation of roasted peanuts, fried peanuts and peanut butter, or ground peanuts into a paste after roasting (Miyagi, 2017; Chiou et al., 1991; Isleib et al., 2008b; Pattee et al., 1994, 1995, 1997, 1998).

In this study, analysis of variance (ANOVA) and genotype + genotype × environment (GGE) biplot were applied to determine the effect of environment, genotype, and G × E interaction in sensory attribute evaluation research. Therefore, the aim of this study was to find the winning genotype(s) under three locations, and which location is an ideal one, as well as to investigate the nature and extent of GEI effects on sensory attributes and biochemical components of tested peanut genotypes. Mean squares and total variance proportion was analyzed by combined analysis of variance. The GGE biplot helped in identifying stable genotypes for specific traits or environments. There is significant GEI effect on sensory attributes and biochemical components of 13 peanut genotypes. The GGE biplot for which-won-where of sensory attributes (**Figure 2**) showed S9 was the best performer at FX and JZ, and L10 was the vertex genotype at WF in crunchiness. S9 was the best genotype at this environment in fineness, sweetness, and overall acceptability, respectively. For Discriminativeness vs. representativeness pattern of the GGE biplot for sensory attributes, the environments were JZ for crunchiness, and FX for fineness, bitterness, and roasted peanutty, while WF for sweetness, off-flavor, and overall acceptability (**Figure 4**).

Regarding GEI in sensory attributes, Isleib et al. found that the sweet flavor exhibited the highest repeatability coefficient through evaluating flavor attributes from the multi-state UPPT (United Peanut Product Testing) over a four-year period (2002-2005) (Isleib et al., 2008b). Pettee et al. also observed the sweet and roasted peanut attributes had little genotype by-year interactions, the breeder could achieve the same level of precision by obtaining the same number of observations in a single year at more locations rather than by testing at fewer locations across more years (Pattee et al., 1997).

Norden et al. evaluated peanut cultivars and germplasm accessions in their Florida breeding program in 1987 and found that genotype significantly affected the O/L ratio (ranging from 0.9 to 35) (Norden et al., 1987). Bomma et al. (2024) conducted multi-environment testing for G×E interactions and identification of high-yielding, stable, medium-duration pigeon pea genotypes by a single year and Esan et al. (2023) analyzed of Bambara groundnut for agronomic performances under three environmental conditions by one year trail. The experimental results are of certain value and can provide references for researchers engaged in related fields. Although only one-year test was conducted in this study, multiple locations were selected, and the locations spanned five latitudes. The two tested locations in Liaoning province, Jinzhou and Fuxin are geographically close, there are significant climatic differences between the two locations (**Table 2**). Fuxin has lower temperatures and less rainfall. While both areas have sandy loam soil, the soil in Fuxin has a higher sand content, and there are also substantial differences in soil nutrients (**Table 3**). These are both major northern peanut-producing regions, and the peanuts produced here are almost free of aflatoxin contamination, which is very important for the safety of sensory evaluation.

The correlations among sweet, bitter, and roasted peanut attributes that were previously reported (Pattee et al., 1998) were also manifested in this study. Specifically, there was a positive correlation between sucrose and sweetness, and there was also a positive correlation with crunchiness and roasted peanutty (**Table 9**). However, this is in contrast to the findings of Oupadissakoon and Young, who reported significant negative correlation between total sugar content and roasted peanut attribute in a limited sample of Virginia-type genotypes (Oupadissakoon and Young, 1984). Correlations of total sugars with the astringent attribute were similar to those with bitterness. Because sucrose accounts for most of the total sugars in the peanut genotypes evaluated, the associations of sucrose with sensory attributes were very similar to those of total sugars.

Mostly related studies identified that HO (high-oleic) peanuts have improved flavors to conventional peanuts due to their resistance to oxidation (Baker et al., 2002; Braddock et al., 1995; Mugendi et al., 1998; Pattee et al., 2002; Riveros et al., 2010), with Nepote et al. reporting that HO peanuts were highly accepted by consumers, and HO peanuts in general were found to have a better flavor quality, which included lower levels of cardboard or oxidized flavors than regular peanuts (Nepote et al., 2009). Only Isleib reported that HO trait not being associated with any changes in flavor or sensory quality (Isleib et al., 2006).

The peanuts used in this study, with the exception of the control variety, are all high oleic peanuts, which have a higher nutritional value, are better for human health and have a longer shelf life. We also found that peanuts with high oleic acid had a crunchier texture, stronger roasted peanutty flavor, sweeter, and overall preferred, which was consistent with the results of the mostly previous researchers. In Argentina, researchers compared high oleic peanuts with normal peanuts for consumer acceptance, oxidation chemical indicators and organoleptic characteristics, moreover screened high oleic peanut lines, like 9399-10, could replace normal peanuts without affecting consumer acceptance of processed peanut products (Nepote et al., 2009). A similar situation also prevailed in our research, a small-seeded high oleic peanut line S9 superior to Huayu20 and a large-seeded high oleic peanut line L10 superior to Huayu33 were screened, both Huayu20 and Huayu33 were widely used as raw materials in the industry. These two lines not only had good sensory quality, but also had good yields. Huayu20 was a Spanish type small-seeded peanut variety with high quality and high yield, it was very popular in the markets, and S9 with better taste are expected to replace it, which is of great significance to peanut processing enterprises in China.

This current study found that not only genotypes, but also locations, accounted for the majority of the total variation in both sensory attributes and biochemical components. The peanuts from Weifang exhibited greater crunchiness, fineness, sweetness, and low bitterness, while also containing higher levels of sucrose, protein, and oleic acid, and lower levels of proanthocyanidin and vitamin E than the peanuts from the other two locations. Climatic data and soil quality are all factors affecting the sensory quality of peanuts. Microclimate data during the growing season was listed in **Table 2**, it can be seen that the temperature in Weifang is more suitable for peanut growth during the peanut growing season. In particular, large-seeded peanuts (Virginia type) have higher temperature requirements. Additionally, rainfall was more sufficient in Weifang, and moisture is also a key factor for ensuring peanut quality. The contents of available potassium, available phosphorus, and nitrogen in the soil of Weifang are significantly higher than that in the other two tested locations (**Table 3**). Combining the above factors, they may be the reasons for superior sensory quality of peanuts cultivated in Weifang.

From the aspect of production, when it comes to the influence on sensory qualities such as crunchiness, off-flavor, and roasted peanutty, genetic factors are more dominant than environmental factors; but for fineness, sweetness, and bitterness, environmental factors are more influential than genetic factors. In respect of the impact on biochemical components, for sucrose and protein content, environmental factors have a greater effect than genetic factors, and for the other components, genetic factors are more important than environmental factors. Through the above combined analysis, in order to breeding peanuts with high protein content and fine texture, as well as peanuts with high sucrose content and sweet taste, it is necessary to pay great attention to the selection of the planting area and cooperate with certain agronomic measures. In the future, being able to breed peanuts with stable sweetness and fineness performance across different locations will represent a significant breakthrough in breeding.

## 5 Conclusions

Sensory quality is one of the most important objectives of peanut breeding program. In this report, influence of location and genotype on sensory quality and biochemical components of peanut was evaluated. Significant differences were observed among genotypes and locations, and the G × E interactions for sensory quality and biochemical components of the tested peanuts. Overall acceptability was significantly positively correlated with crunchiness, fineness, sweetness, roasted peanutty and significantly negatively correlated with bitterness for roasted peanuts. In addition, there is an obvious correlation between the biochemical components of peanut and the sensory quality. Among them, peanuts with high oleic acid were overall preferred, had a crunchier texture, were sweeter, and had a stronger roasted peanutty flavor, while peanuts with high oil were more delicate, less sweet and less roasted peanutty flavor, and peanuts with high protein were also more delicate, were sweeter and stronger roasted peanutty flavor. Moreover, the proanthocyanidin content is significantly positively correlated with bitterness and off-flavor, and is negatively correlated with crunchiness, fineness, sweetness and overall acceptability. Furthermore, peanuts with high Vitamin E had a relatively less intense roasted peanutty flavor. Two peanut lines S9 and L10 were screened with high oleic and better sensory quality, which can be used in peanut breeding program for improving the sensory quality. Weifang as a peanut-planting area, it can produce high sensory quality peanuts for the peanut processing enterprises. This data can serve as a reference for peanut planting regions with the same latitude or similar climatic conditions.

## Data Availability

The raw data supporting the conclusions of this article will be made available by the authors, without undue reservation.
